# Book Reviews

**Published:** 1901-05

**Authors:** 


A System of Practical Therapeutics. By Eminent American and Foreign Authorities. Edited by Hobart Amory Hare, M. D., Professor of Therapeutics, Jefferson Medical College; Physician to Jefferson College Hospital, etc., Philadelphia. New (2d) edition, thoroughly revised. In three octavo volumes, containing 2593 pages, with 427 engravings, and 26 full-page colored plates. Philadelphia and New York: Lea Brothers & Co. 1901. [Price, per volume, cloth, $5.00; leather, $6.00; half morocco, $7.00.]

Two volumes of this excellent system of therapeutics have already been reviewed in this journal (April, 1901.) It is with pleasure that we present our readers with a brief comment on the third volume that is devoted to surgical treatment. Its contents include the following:

Anesthesia and anesthetics (new), by Charles Lester Leonard; Surgical technique (new), by Charles H. Frazier; The treatment of fractures and dislocations (new), by Henry R. Wharton; Minor surgery and bandaging (new), by George W. Spencer; Cerebral concussion and shock, Joseph Ransohoff; Pleural effusion and empyema, abscess and gangrene of the lung, by A. J. McCosh; Peritonitis, non-operative and post-operative, appendicitis, paratyphlitic abscess and obstruction of the bowels, by George Ryerson Fowler; Obstruction of the intestines, by Edward Martin; Diseases of the rectum and anus, by Joseph M. Mathews; Therapeutics of the male genito-urinary tract, by William T. Belfield; Therapeutics of the genito-urinary diseases of women, by Edward E. Montgomery; Therapeutics of pregnancy, parturition and the puerperal state (new), by Edward P. Davis; Diseases of the eye and their treatment by the general practitioner, by Casey A. Wood; Diseases of the ear and their treatment by the general practitioner, by S. MacCuen Smith; Diseases of the nasal chambers and associated affections, by E. Fletcher Ingals; Diseases of the uvula, the pharynx and larynx, by D. Braden Kyle.

Even a cursory inspection of the foregoing list of subjects and authors, will convince the experienced physician that this volume contains just such information as the general practitioner of medicine needs when, as is always likely to happen, he is called upon to do surgical work, and especially to conduct post-operative management.

We have been interested especially in the chapters on anesthesia, surgical technique, fractures, minor surgery, peritonitis and appendicitis, obstruction of the intestines and diseases of the rectum. Each one of these contains much of a practical character that confronts a physician almost every day. The volume, too, is well illustrated; in particular are Dr. Fowler's plates, showing each step in the operation for appendicitis, and the illustrations of minor surgery are to be commended for clearness and artistic excellence.

The system, as a whole, is one of the most important additions a busy physician can make to the working literature of his library.

Introduction to the Study of Medicine. By G. H. Roger, Professor Extra ordinary in the Faculty of Medicine of Paris; Member of the Biological Society; Physician to the Hospital of Porte D'Aubervilliers. Authorised translation by M. S. Gabriel, M. D., with additions by the author. Octavo, pp. 551. New York: D. Appleton & Co. 1901. [Price, cloth, $5 00; sheep, $6.00.]

This work is constructed on entirely new lines of medical teaching, at least as far as this country is concerned. It is a reproduction of a course of lectures delivered at the University of Paris, the object of which was to teach students how to begin the study of medicine, how to economise time, and what books to read. Professor Roger is an interesting teacher; he adopts a simple plan and uses simple language in its explanation. He unfolds the object of medicine as a science and an art, tells by what means it may be studied, explains how one becomes sick, and then proceeds to the consideration of morbific influences that constantly threaten the stability of health.

The distinguished author devotes 200 or more pages to the examination of the sick as a distinct and separate subject, every page of which is full of useful information, and all may be read with profit by every physician. While Roger does not belittle the importance of experimental pathology he advances clinical investigations to their true place, not second to but abreast of the former, in the interpretation of the language of disease.

The lectures embrace every known element in the causation of disease and its cure. They deal with mechanical, physical, chemical and animate agents; with the etiology of infections and the pathogenesis of infectious diseases; with nervous reactions, disturbances of nutrition, and autointoxications; with heredity, inflammation, and septicemia; with tumors, cellular degeneration, functional synergies and morbid sympathies; with evolution of diseases, clinical application of scientific procedures, diagnosis, prognosis and therapeutics. These and many other factors in disease and cure are discoursed upon with clearness, and the author follows step by step the morbid processes, avoiding mere theories, but reporting results that appear to be final.

It is difficult to classify this work, because it is so unique as to be *sui generis.* It is, in the main, a book that may be read with better understanding by the advanced student, but may justly be considered a contribution to high class medical literature.

PHYSICAL DIAGNOSIS IN OBSTETRICS A guide in Antepartum, Partum and Postpartum Examinations for the Use of Physicians and Undergraduates. By EDWARD A AYERS, M D, Professor of Obstetrics in the New York Polyclinic; Attending Physician to the Mothers and Babies Hospital. Octavo, pp. 283. Illustrated. New York: E. B. Treat & Co. 1901. [Price, $2.00.]

The author of this book, who has taught clinical obstetrics for eighteen years, aims to present in book form, more fully and more carefully than can be done in the clinic room, a clinical study of obstetrics, as he has pursued it in giving verbal instruction. How well he has accomplished his purpose only those, perhaps, who listened to him at the bedside can determine; but it is quite true that the teaching of obstetrics has undergone radical changes the past few years, and the author has done well in his attempt to keep literature apace with clinical instruction.

The title of his book is somewhat unique, and appeals to the modern taught obstetrician. Why should not "physical diagnosis," which is so rigidly insisted upon by authors on diseases of the chest, be applied with equal insistence to the phenomena of pregnancy and the parturient? This work is a most useful supplement to the obstetric text-book, it contains much of value that the latter does not, and will assist beginners in obstetric practice to solve many doubtful problems. Moreover, it is essentially a scientific treatise and so will aid the instructor and practitioner in their daily teachings and obstetric work.

---

EXPERIMENTAL RESEARCH INTO THE SURGERY OF THE RESPIRATORY SYSTEM. An Essay awarded the Nicholas Senn prize by the American Medical Association for 1898. By GEORGE W. CRILE, A.M., M.D. Ph, D., Professor of Clinical Surgery, Western Reserve University; Surgeon to St. Alexis Hospital; Associate Surgeon to Lakeside Hospital, Cleveland. Octavo, pp. 114. Second edition. Philadelphia: J. P. Lippincott Co. 1900.

The author of this essay is a practical surgeon as well as a skilful worker in experimental fields of research. It is quite unusual to find the two elements of science so happily combined. Too often the prize essayist is only a theorist. The researches detailed in this book extended over a period of two years, and were conducted upon dogs. The aim has been to dwell upon the most practical side of the several phases of the subject dealt with, which is a most commendable plan to pursue.

The surgery of the respiratory system is of constantly increasing interest as it becomes better understood, and the work of this author will contribute not a little to a clearer understanding of doubtful points. The effects of inhaling hot air and flame, the cause of collapse or death from blows upon the lower chest and epigastrium, and foreign bodies in the upper-air tract are especially of interest and are well considered. The prize was worthily bestowed and surgeons will eagerly buy the book.

